# Reviewers

**DOI:** 10.1111/cas.70314

**Published:** 2026-01-06

The publication of invaluable papers in Cancer Science depends on the prompt, careful review of submitted manuscripts. We would like to thank the following experts for reviewing manuscripts submitted between October 1, 2024 to September 30, 2025.

Hiroyuki Abe

Tatsuya Abe

Toshiya Abe

Jun Adachi

Shungo Adachi

Ahmad Adawy

Hisaki Aiba

Masahiko Ajiro

Shusuke Akamatsu

Yoshiki Akatsuka

Jun Akiba

Hirofumi Akita

Toru Akiyama

Yoshimitsu Akiyama

Joseph Amankwaah

Takahiro Ando

Samuel Antwi

Kosuke Aoki

Shigeki Aoki

Shuichi Aoki

Seiji Arai

Hisashi Arase

Tomohiro Arita

Yuki Arita

Ken Asada

Shuhei Asada

Ryutaro Asano

Kotaro Azuma

Masaya Baba

Tsukasa Baba

Hiroko Bando

Narikazu Boku

Eric Bouffet

Horacio Cabral

Junchao Cai

Kun Cai

Luis G. Carvajal‐Carmona

Peter H. Cashin

Natsuko Chiba

Shigeru Chiba

Tatsuyuki Chiyoda

Roberto Coppo

Adam P. Cribbs

Kaisa Cui

Haruko Daga

Shipeng Dai

Shingo Dan

Dajun Deng

Hidetoshi Eguchi

Mariko Eguchi

Shogo Ehata

Itaru Endo

Makoto Endo

Motoyoshi Endo

Tomohiro Enokida

Vincent Fradet

Juan Fueyo

Shigeo Fuji

Hiroyuki Fujii

Satoshi Fujii

Shin‐Ichiro Fujii

Ayumi Fujimoto

Daichi Fujimoto

Atsushi Fujimura

Takao Fujisawa

Teruaki Fujishita

Hiroaki Fujita

Kazutoshi Fujita

Yukio Fujiwara

Yutaka Fujiwara

Tomohiko Fukuda

Keisuke Fukui

Manabu Fukumoto

Hiroshi Fukushima

Itoh Fumiko

Shinsuke Funakoshi

Yuji Funakoshi

Chikako Funasaka

Michinori Funato

Kyoji Furukawa

Tatsuhiko Furukawa

Toru Furukawa

Mitsuru Futakuchi

Morteza Gholami

John Glod

Akiteru Goto

Hidemasa Goto

Osamu Gotoh

Jichun Gu

Shashi Gujar

Haochen Guo

Zhen Guo

Zhongliang Guo

Hiroshi Haeno

Yoshimi Haga

Shotaro Hagiwara

Akinobu Hamada

Yasuo Hamamoto

Junzo Hamanishi

Hirofumi Hanaoka

Rikinari Hanayama

Hiroshi Handa

Masatoshi Hara

Tomoaki Hara

Hiroshi Harada

Yohei Harada

Daigo Hashimoto

Itaru Hashimoto

Naoya Hashimoto

Shinichi Hashimoto

Taiki Hashimoto

Hisashi Hasumi

Kenji Hata

Shigetsugu Hatakeyama

Shingo Hatakeyama

Koji Hatano

Yoshihiro Hatta

Kazuki Hattori

Naoko Hattori

Fumihiko Hayakawa

Yoku Hayakawa

Shinya Hayami

Koichi Hayano

Hidetoshi Hayashi

Hideyuki Hayashi

Takuo Hayashi

Tsutomu Hayashi

Yoshihiro Hayashi

Eva Hernando

Zoltan Herold

Yoichi Hiasa

Susumu Hijioka

Hayato Hikita

Susumu Hirabayashi

Toru Hiraga

Hideyo Hirai

Masaki Hirano

Shigeru Hirano

Nobuyoshi Hiraoka

Akira Hirasawa

Eishu Hirata

Hidenari Hirata

Kenji Hirata

Kenro Hirata

Yoshihiko Hirohashi

Sekine Hiroki

Yuichi Hisamatsu

Asahi Hishida

Tsunaki Hongu

Koichi Honke

Toshiyuki Hori

Kuniko Horie

Rie Horii

Hidehito Horinouchi

Keisuke Horiuchi

Daisuke Hoshi

Takayuki Hoshii

Ayuko Hoshino

Waki Hosoda

Yasuyuki Hosono

Noriko Hosoya

Miller Huang

Zhenkun Huang

Eiki Ichihara

Daisuke Ichikawa

Shintaro Ichikawa

Tomonaga Ichikawa

Masashi Idogawa

Kenji Iemura

Kazuhiko Igarashi

Keita Iida

Yuichi Iida

Hisashi Iizasa

Hideaki Ijichi

Hideki Ikeda

Kazuhiro Ikeda

Satoshi Ikeda

Masachika Ikegami

Hiroki Imada

Yusuke Imagawa

Yoichi Imai

Kentaro Inamura

Daichi Inoue

Takahiro Inoue

Yasumichi Inoue

Osamu Ishibashi

Masato Ishigami

Seiichiro Ishihara

Akira Ishikawa

Eiichi Ishikawa

Hideki Ishikawa

Hitoshi Ishikawa

Toru Ishikawa

Yuichi Ishikawa

Takatsugu Ishimoto

Takamasa Ishino

Kenji Ishitsuka

Toshiyuki Ishiwata

Md. Mohaimenul Islam Rubel

Hideko Isozaki

Kota Itahashi

Hidemi Ito

Shigeki Ito

Takahiro Ito

Yuta Ito

Shinji Itoh

Susumu Itoh

Toshiki Itoh

Yu Iwabuchi

Masao Iwagami

Moriya Iwaizumi

Atsushi Iwama

Eiji Iwama

Takeshi Iwasaki

Takashi Iwata

Masaaki Iwatsuki

Takeshi Iwaya

Koji Izutsu

Sankar Jagadeeshan

Yebin Jiang

Yifan Jiang

Minji Jo

Yoshikazu Johmura

Todd Johnson

Hiroshi Kagamu

Hidenori Kage

Shun‐Ichiro Kageyama

Atsushi Kaida

Daisuke Kaida

Hiroaki Kajiho

Taisuke Kajino

Yoshihiro Kakeji

Takeshi Kamakura

Takashi Kamatani

Osamu Kaminuma

Masashi Kanai

Masayuki Kanamori

Masatoshi Kanayama

Hiroaki Kanda

Shuzo Kaneko

Gyeong Hoon Kang

Kennosuke Karube

Hiroaki Kasashima

Shinichiro Kashiwagi

Hiromi Kataoka

Hiroyuki Katayama

Kazuhiro Katayama

Yuki Katayama

Minoru Kato

Motohiro Kato

Shota Kato

Taigo Kato

Yasutaka Kato

Hiroto Katoh

Keisuke Katsushima

Eriko Katsuta

Hiroo Katsuya

Asuka Kawachi

Manabu Kawada

Kosuke Kawaguchi

Kohichi Kawahara

Taketo Kawai

Akinori Kawakami

Eiryo Kawakami

Hisato Kawakami

Daisuke Kawakita

Shimpei Kawamoto

Kei Kawana

Shinpei Kawaoka

Yoshihiro Kawasaki

Atsunari Kawashima

Makoto Kawatani

Akihito Kawazoe

Masahito Kawazu

Evan Keller

Hirotsugu Kenmotsu

Hiroyasu Kidoya

Eiji Kikuchi

Yoshinao Kikuchi

Yoshikane Kikushige

Hirokazu Kimura

Takahiro Kimura

Ichiro Kinoshita

Masashi Kinoshita

Akihiro Kitadate

Hiroshi Kitajima

Shunsuke Kitajima

Hidemitsu Kitamura

Hiroshi Kitamura

Akira Kitanaka

Naomi Kiyota

Kazuma Kiyotani

Hiroki Kobayashi

Hiroshi Kobayashi

Hiroya Kobayashi

Hisataka Kobayashi

Shogo Kobayashi

Takashi Kobayashi

Yoshihisa Kobayashi

Yuta Kobayashi

Yuta Kochi

Takahiro Kodama

Fumitaka Koga

Hironori Koga

Yoshikatsu Koga

Kentaro Kogure

Yasunori Kogure

Kenichi Kohashi

Tomohiro Kohmoto

Susumu Kohno

Motohiro Kojima

Takashi Kojima

Yasuhiro Kojima

Yasushi Kojima

Victor C. Kok

Yu‐Ichiro Koma

Masaaki Komatsu

Keigo Komine

Daisuke Komura

Jumpei Kondo

Satoru Kondo

Sun‐Young Kong

Tim Kong

Akimitsu Konishi

Hirotaka Konishi

Masamitsu Konno

Chihaya Koriyama

Kazuto Kosaka

Naohiko Koshikawa

Fujimoto Kosuke

Norihiro Kotani

Masashi Koto

Nobuji Kouno

Yuriko Koyanagi

Toshiyuki Kozuki

Shuji Kubo

Terufumi Kubo

Yasutoshi Kuboki

Takahiro Kuchimaru

Yasusei Kudo

Yuji Kuge

Iwao Kukimoto

Shogo Kumagai

Kensuke Kumamoto

Kei Kunimasa

Alvin Guo Kunyao

Chihhsi Kuo

Makoto Kurano

Kozo Kuribayashi

Ryota Kurimoto

Junya Kuroda

Masahiko Kuroda

Koji Kurose

Kosuke Kusamori

Shigeru Kusumoto

So Hee Kwon

Brent Larson

Pedro Lazo

Ke Lei

Hao Li

Lianbo Li

Yi Li

Zhuan Li

Chao Liu

Haiou Liu

Jinhui Liu

Qiang Liu

Wen‐Cai Liu

Yi Lu

Peng Luo

Artem Lysenko

Xiaoming Lyu

Mitsuhiro Machitani

Yurina Maeshima

Nako Maishi

Hideki Makinoshima

Shinichi Makita

Yingying Mao

Tasuku Mariya

Yoshiaki Maru

Junichi Maruyama

Tetsuo Mashima

Ken Masuda

Kiyoshi Masuda

Mari Masuda

Takaaki Masuda

Mitsuko Masutani

Daisuke Matsubara

Satoru Matsuda

Hirotaka Matsui

Hiroki Matsumoto

Hiromi Matsumoto

Koji Matsumoto

Kunio Matsumoto

Shingo Matsumoto

Shinji Matsumoto

Tomonori Matsumoto

Yoshihisa Matsumoto

Noriomi Matsumura

Yoshihiro Matsumura

Hirotami Matsuo

Akira Matsushita

Kazuyuki Matsushita

Kazuto Matsuura

Atsushi Matsuzawa

Reiko Matsuzawa

Franz Meitinger

Toshi Menju

Shinji Mii

Hirokazu Miki

Naoya Mimura

Yoji Minamishima

Yuji Minegishi

Eikan Mishima

Natalia V. Mitiushkina

Yoichiro Mitsuishi

Masahiko Miura

Masatomo Miura

Satoru Miura

Yuji Miura

Yohei Miyagi

Yuki Miyai

Makito Miyake

Kanako Miyano

Minoru Miyashita

Kenichi Miyata

Eisaku Miyauchi

Makoto Miyazaki

Hiroaki Miyoshi

Yasuo Miyoshi

Yusuke Mizukami

Kazuyuki Mizuno

Raphael Moeckli

Isao Momose

Yukihide Momozawa

Masato Morikawa

Akira Morimoto

Masahiro Morise

Shinichiro Motohashi

Noriko Motoi

Ken‐Ichi Mukaisho

Kazuhiro Murakami

Kosuke Murakami

Ryusuke Murakami

Yuichi Murakami

Yasuhiro Murakawa

Daisuke Muraoka

Kenji Murata

Shigeo Murata

Tomoya Muto

Michihiro Mutoh

Genta Nagae

Koji Nagafuji

Masayuki Nagahashi

Hirokazu Nagai

Osamu Nagano

Koji Nagaoka

Hiroaki Nagashima

Satoshi Nagayama

Taku Naiki

Yoichi Naito

Yutaka Naito

Kazuhito Naka

Hayato Nakagawa

Masahiro Nakagawa

Masao Nakagawa

Eijiro Nakamura

Katsumasa Nakamura

Kenichi Nakamura

Kohei Nakamura

Takafumi Nakamura

Takashi Nakamura

Yuki Nakanishi

Hiroyasu Nakano

Takashi Nakaoku

Kayo Nakata

Hironao Nakayama

Joji Nakayama

Jun Nakayama

Masanori Nakayama

Mizuho Nakayama

Kenjiro Namikawa

Shigeki Nanjo

Yasuhito Nannya

Shintaro Narita

Koji Nata

Manabu Natsumeda

Takahide Nejo

Naoe Nihira

Seiji Niho

Atsushi Niida

Satoshi Ninagawa

Kiichiro Ninomiya

Kouki Nio

Chinatsu Nishida

Jun Nishida

Mikako Nishida

Naohiro Nishida

Momoko Nishikori

Hiroshi Nishina

Takashi Nishina

Michiru Nishita

Hideki Nishitoh

Satoshi Nishiwaki

Naoyuki Nishiya

Satoshi Nishizuka

Sumihito Nobusawa

Takehiro Noda

Rei Noguchi

Takahiro Nomoto

Kaname Nosaki

Hiroaki Nozawa

Junna Oba

Kazutaka Obama

Mieko Ochi

Toshiki Ochi

A Ogawa

Kazuma Ogawa

Mikako Ogawa

Hirokazu Ogino

Takayuki Ogino

Tetsuya Oguri

Takashi Ohama

Yuuki Ohara

Akihiro Ohashi

Kadoaki Ohashi

Ryuji Ohashi

Shigeo Ohba

Tomokazu Ohishi

Yuki Ohkawa

Takayuki Ohkuri

Akihiro Ohmoto

Tatsuya Ohno

Masayuki Ohtsuka

Takahiro Oike

Satoshi Oizumi

Hidenori Ojima

Masahiro Oka

Hitoshi Okada

Tetsuya Okajima

Akihiko Okamura

Susumu Okano

Shohei Okazaki

Toshihiko Okazaki

Yukari Okita

Yoichiro Okubo

Kaoru Okugawa

Yoshinaga Okugawa

Yusuke Okuma

Kazuhiro Okumura

Yusuke Okuno

Urszula Oleksiewicz

Chitose Oneyama

Rikiya Onimaru

Hiroaki Ono

Makiko Ono

Ichiro Onoyama

Takumi Onoyama

Kunishige Onuma

Akira Orimo

Makoto Osanai

Hiroko Oshima

Toshinori Ozaki

Yuki Ozato

Isao Oze

Cheng Pan

Amanda Panfil

Jae‐Hyun Park

Woongyang Park

Samuel Pena‐Llopis

Jesus Perez‐Losada

Shuai Ping

Jens T. Van Praet

Lin Qi

Xiaowei Qi

Wei Qin

Xian‐Yang Qin

Shinya Rai

Vijay Ramaswamy

Sherif Rashad

Masaki Ri

Susumu Rokudai

Ryota Sada

Daichi Sadato

Sumito Saeki

Ryo Saga

Daisuke Saigusa

Ken Saijo

Yuriko Saiki

Motonobu Saito

Shoji Saito

Takuro Saito

Yasuhiro Saito

Yasuyuki Saito

Yoshinobu Saito

Yuki Saito

Masao Saitoh

Masakiyo Sakaguchi

Katsuya Sakai

Kazuko Sakai

Takehiko Sakai

Tetsuya Sakai

Jun Sakakibara‐Konishi

Tomoki Sakakida

Naoya Sakamoto

Shinichi Sakamoto

Shuichi Sakamoto

Shingo Sakashita

Tomohisa Sakaue

Yukimi Sakoda

Fuminori Sakurai

Hiroaki Sakurai

Oltea Sampetrean

Masashi Sanada

Takaomi Sanda

Katsuhiro Sano

Eri Sasabe

Yoji Sasahara

Atsuo Sasaki

Atsuo T Sasaki

Masaoki Sasaki

Motoko Sasaki

Nobunari Sasaki

So‐Ichiro Sasaki

Takaaki Sasaki

Yuka Sasaki

Goro Sashida

Eiichi Sato

Kuniaki Sato

Mitsuo Sato

Naru Sato

Shinya Sato

Takahiko Sato

Tatsuhiro Sato

Waka Sato

Yasuharu Sato

Yusuke Sato

Taroh Satoh

Takeshi Sawada

Yu Sawada

Nita Seibel

Miho Sekai

Masau Sekiguchi

Kentaro Semba

Hubing Shi

Tongguo Shi

Ryota Shibaki

Nobuhiro Shibata

Yuji Shibata

Kazuko Shibuya

Kazuhiko Shien

Daisuke Shigemi

Kazuyuki Shimada

Shu Shimada

Hideyuki Shimizu

Kouhei Shimizu

Seiichi Shimizu

Asako Shimoda

Kazuya Shimoda

Masayuki Shimoda

Tatsunori Shimoi

Akihiko Shimomura

Aesun Shin

Keiko Shinjo

Daisuke Shiokawa

Masaki Shiota

Masayuki Shirasawa

Hidekazu Shirota

Katsutoshi Shoda

Siro Simizu

Deo R. Singh

Rakesh K. Singh

Yasushi Soda

Kenbun Sone

Yukihiko Sonoda

Guillaume St‐Jean

Chunxia Su

Kenichi Suda

Yasuhito Suehara

Yutaka Suehiro

Tsuyoshi Sugiura

Daisuke Sugiyama

Eri Sugiyama

Hiromi Sugiyama

Hiroshi Sugiyama

Shigeaki Sunada

Yu Sunakawa

Kazuhito Suziki

Ayako Suzuki

Chiaki Suzuki

Hiromichi Suzuki

Hiroshi I. Suzuki

Hiroyuki Suzuki

Miho Suzuki

Rei Suzuki

Shiro Suzuki

Shugo Suzuki

Takafumi Suzuki

Takehiro Suzuki

Takeshi Suzuki

Masatoshi Tagawa

Yoshiyuki Tagayasu

Megumi Tago

Ayumi Taguchi

Ayumu Taguchi

Kazuhiro Taguchi

Keiko Taguchi

Satoru Taguchi

Masatoshi Takagi

Satoshi Takagi

Takayuki Takahama

Miki Takahara

Masanobu Takahashi

Naoto Takahashi

Satoshi Takahashi

Tsuyoshi Takahashi

Atsushi Takai

Hiroyuki Takamatsu

Manabu Takamatsu

Akiyoshi Takami

Shiki Takamura

Shinichi Takano

Atsushi Takatori

Kenichi Takayama

Kazuyoshi Takeda

Masashi Takeda

Masayuki Takeda

Tetsuo Takehara

Hisanori Takenobu

Fukumoto Takeshi

Hideyuki Takeshima

Hiroya Takeuchi

Masashi Takeuchi

Yasuto Takeuchi

Shuji Takiguchi

Keiyo Takubo

Motohiro Tamiya

Yoichiro Tamori

Kazuo Tamura

Ryota Tamura

Tomohiko Tamura

Kezhe Tan

Masahiko Tanabe

Atsushi Tanaka

Hajime Tanaka

Kousuke Tanaka

Nobuyuki Tanaka

Noritaka Tanaka

Yosuke Tanaka

Fuzhou Tang

Masaji Tani

Tomohiko Taniai

Hiroya Taniguchi

Kohei Taniguchi

Koji Taniguchi

Makoto Taniguchi

Chizu Tanikawa

Junko Tanizaki

Rikiya Taoka

Kundu Tapas

Roberta Tarallo

Yusuke Tarumoto

Etsu Tashiro

Kensuke Tateishi

Ryosuke Tateishi

Shotaro Tatekawa

Hiro Tatetsu

Daisuke Tatsuda

Isao Tawara

Hiroshi Tazawa

Teh Bin Tean

Naoki Terada

Hideki Terai

Koji Teramoto

Takeshi Terashima

Yasuhito Terui

Fredrik Ivar Thege

Yoshihisa Tokumaru

Eriko Tokunaga

Fuminori Tokunaga

Akihiro Tomita

Daisuke Tomizawa

Shinichi Toyooka

Kosuke Tsuchiya

Naoto Tsuchiya

Masumi Tsuda

Masataka Tsuge

Takahiro Tsujikawa

Masatsune Tsujioka

Tomohide Tsukahara

Hirotake Tsukamoto

Tetsuya Tsukamoto

Toyonori Tsuzuki

Ryosuke Uchibori

Keiji Uchiyama

Keiko Udaka

Yutaka Ueda

Satoshi Ueha

Ryota Uehara

Arisa Ueki

Motohide Uemura

Takayuki Ueno

Tomotaka Ugai

Shigeki Umemura

Hae Ung

Kaname Uno

Fumihiko Urabe

Genki Usui

Hirokazu Usui

Yoshiaki Usui

Lene H. S. Veiga

Keiko Wada

Satoshi Wada

Fu Wang

Satoshi Watanabe

Gaofei Wei

Liang Weng

Yong‐Bing Wu

Qipeng Xie

Kailin Xu

Song Xu

Hiroshi Yagi

Tomonori Yaguchi

Rin Yamada

Tadaaki Yamada

Takeshi Yamada

Yosuke Yamada

Hirofumi Yamaguchi

Junya Yamaguchi

Kiyoshi Yamaguchi

Motoko Yamaguchi

Taiki Yamaji

Gaku Yamamoto

Keita Yamamoto

Masahiro Yamamoto

Masato Yamamoto

Mizuki Yamamoto

Seiichiro Yamamoto

Yusuke Yamamoto

Tomohiko Yamane

Fumiyuki Yamasaki

Riu Yamashita

Satoshi Yamashita

Taro Yamashita

Tatsuya Yamashita

Motohiro Yamauchi

Nobuhiko Yamauchi

Shota Yamauchi

Takahiro Yamauchi

Asako Yamayoshi

Etsuko Yamazaki

Masaya Yamazaki

Jun Yan

Teruki Yanagi

Hideyuki Yanai

Hiroyuki Yanai

Wen Yang

Xi Yang

Shuya Yano

Masakazu Yashiro

Hiroyuki Yasuda

Hironobu Yasui

Jun‐Ichirou Yasunaga

Mujie Ye

Satomi Yogosawa

Takehiko Yokobori

Akira Yokoi

Akihiko Yokoyama

Yasuhisa Yokoyama

Ryoma Yoneda

Kimio Yonesaka

Emiko Yoshida

Hiroshi Yoshida

Kenichi Yoshida

Kosuke Yoshida

Masayuki Yoshida

Tatsuya Yoshida

Kosuke Yoshihara

Tetsuro Yoshimaru

Yasuhiro Yoshimatsu

Koji Yoshimoto

Kiyoshi Yoshimura

Yusuke Yoshioka

Hironori Yoshiyama

Akihiko Yoshizawa

Ochi Yotaro

Mahmoud Younis

Zhuo Yu

Junichiro Yuda

Mayu Yunokawa

Yoshitaka Zenke

Congcong Zhang

Zhigang Zhang

Gang Zhao

Shukang Zhao

Zhijie Zhao

Binyan Zhong

Chongchang Zhou

Rongrong Zhou

Shuyan Zhou

Xia Zhu

Yuan Zhuang

